# Identification and Map-Based Cloning of the Light-Induced Lesion Mimic Mutant 1 (*LIL1*) Gene in Rice

**DOI:** 10.3389/fpls.2017.02122

**Published:** 2017-12-19

**Authors:** Qian Zhou, Zhifei Zhang, Tiantian Liu, Bida Gao, Xingyao Xiong

**Affiliations:** ^1^Hunan Provincial Key Laboratory for Biology and Control of Plant Diseases and Insect Pests, College of Plant Protection, Hunan Agricultural University, Changsha, China; ^2^Hunan Provincial Key Laboratory for Germplasm Innovation and Utilization of Crop, Changsha, China; ^3^Agricultural College, Hunan Agricultural University, Changsha, China

**Keywords:** lesion mimic mutant (LMM), hypersensitive response (HR), map-based cloning, rice (*Oryza sativa* L.), rice blast resistance

## Abstract

The hypersensitive response (HR) is a mechanism by which plants prevent the spread of pathogen. Despite extensive study, the molecular mechanisms underlying HR remain poorly understood. Lesion mimic mutants (LMMs), such as *LIL1* that was identified in an ethylmethane sulfonate mutagenized population of *Indica* rice (*Oryza sativa* L. ssp. *Indica*) 93-11, can be used to study the HR. Under natural field conditions, the leaves of *LIL1* mutant plants exhibited light-induced, small, rust-red lesions that first appeared at the leaf tips and subsequently expanded throughout the entire leaf blade to the leaf sheath. Histochemical staining indicated that *LIL1* lesions displayed an abnormal accumulation of reactive oxygen species (ROS) and resulted from programmed cell death (PCD). The *LIL1* mutants also displayed increased expression of defense-related genes and enhanced resistance to rice blast fungus (*Magnaporthe grisea*). Genetic analysis showed that mutation of *LIL1* created a semi-dominant allele. Using 1,758 individuals in the F_2_ population, *LIL1* was mapped in a 222.3 kb region on the long arm of chromosome 7. That contains 12 predicted open reading frames (ORFs). Sequence analysis of these 12 candidate genes revealed a G to A base substitution in the fourth exon of LOC_Os07g30510, a putative cysteine-rich receptor-like kinase (CRK), which led to an amino acid change (Val 429 to Ile) in the LIL1 protein. Comparison of the transcript accumulation of the 12 candidate genes between *LIL1* and 93-11 revealed that LOC_Os07g30510 was up-regulated significantly in *LIL1*. Overexpression of the LOC_Os07g30510 gene from *LIL1* induced a *LIL1*-like lesion phenotype in Nipponbare. Thus, *LIL1* is a novel LMM in rice that will facilitate the further study of the molecular mechanisms of HR and the rice blast resistance.

## Introduction

Plants combat pathogens with a sophisticated immune system consisting of two branches. The first uses transmembrane pattern recognition receptors (PRRs) to recognize conserved pathogen-associated molecular patterns (PAMPs) and activate PAMP-triggered immunity (PTI). The second uses nucleotide binding site-leucine rich repeat (NBS-LRR) proteins to recognize effectors produced by the pathogens and activate effector triggered immunity (ETI). PTI and ETI elicit similar defense responses with ETI more frequently accompanied by a hypersensitive response (HR) (Jones and Dangl, 2006). A defining feature of strong, local defenses associated with the HR is programmed cell death (PCD) of host plant cells. Other events correlated with the HR include ion fluxes, active oxygen bursts, defense-related gene expression, accumulation of antimicrobial compounds and cell wall fortifications (Yin et al., 2000). The HR also activates a whole plant, long-lasting, broad-spectrum resistance to subsequent pathogens infection, referred to as systemic acquired immunity (SAR) (Hunt et al., 1996).

The HR is considered one of the most important reactions of plant resistance to pathogens (Tomiyama, 1963). However, the molecular mechanism regulating the HR remain mysterious. An efficient way to study the molecular mechanisms of HR and disease resistance is to use lesion mimic mutants (LMMs), which exhibit spontaneous, disease-like lesions without pathogen attack. These lesions, which can resemble either disease symptoms or pathogen-inducible HR, frequently are associated with enhanced plant disease resistance to a wide range of pathogens (Lorrain et al., 2003). Numerous LMMs have been identified in many plants, including Arabidopsis (Dietrich et al., 1994; Bowling et al., 1997; Mou et al., 2000), maize (Walbot, 1991; Johal et al., 1995), wheat (Yao et al., 2009; Du et al., 2014; Wang et al., 2016), barley (Wolter et al., 1993; Rostoks et al., 2006) and rice (Takahashi et al., 1999; Yin et al., 2000; Mizobuchi et al., 2002; Campbell and Ponald, 2005; Mori et al., 2007; Wu et al., 2008; Qiao et al., 2010; Chen et al., 2012; Fekih et al., 2015; Li et al., 2016). The notion that complex and diverse pathways underlie the phenotypes of LMMs is supported by the mutated genes falling into many different functional groups, including membrane-associated proteins (Büschges et al., 1997; Lorrain et al., 2004; Noutoshi et al., 2006), ion channel proteins (Balague et al., 2003; Rostoks et al., 2006), an U-Box/Armadillo repeat protein (Zeng et al., 2004), a splicing factor 3b subunit 3 (Chen et al., 2012), a zinc-finger protein (Dietrich et al., 1997), a heat stress transcription factor (Yamanouchi et al., 2002), a clathrin-associated adaptor protein (Qiao et al., 2010) and proteins involved in the biosynthesis and metabolic pathways of phenolic compounds (Gray et al., 1997), such as porphyrin (Hu et al., 1998; Ishikawa et al., 2001) and fatty acids or lipids (Kachroo et al., 2001; Brodersen et al., 2002). Although the phenotypes of some LMMs result from disorder in pathways unrelated to defense responses (Hu et al., 1998; Yamanouchi et al., 2002), extensive studies of other LMMs and their genes has shed light on complex and diverse pathways regulating both the initiation and containment of HR-associated cell death in plants (Lorrain et al., 2003).

In this study, we cloned the gene for which mutation and over-expression in the light-induced lesion mimic mutant 1 (*LIL1*) caused necrotic spots around 3-leaf stage and significantly reduced plant height. Diaminobenzidine (DAB) and trypan blue staining analysis revealed that ROS accumulation and death of plant cells, respectively, accompanied the lesions in the *LIL1* mutant. Meanwhile, we also found that *LIL1* plants had elevated expression of defense-related genes and increased resistance to *Magnaporthe grisea*. Genetic analysis of *LIL1* indicated that the lesion phenotype is controlled by a semi-dominant gene located in a 222.3 kb interval of chromosome 7 that contains 12 predicted open reading frames (ORFs). Sequencing of these ORFs identified a missense mutation from G to A in the fourth exon of a predicted CRK, LOC_Os07g30510, that converts Val 429 to Ile within the serine/threonine protein kinase catalytic (STKc) domain. The expression levels of the 12 ORFs within the cloning interval were also compared between *LIL1* and 93-11 and LOC_Os07g30510 was up-regulated significantly in *LIL1*. Notably, over-expression of the LOC_Os07g30510 gene caused Nipponbare to exhibit a lesion mimic phenotype similar to *LIL1*. Our identification of a novel LMM in a CRK gene, *LIL1*, in rice will facilitate further study of the molecular mechanisms of HR and rice blast resistance.

## Materials and Methods

### Plant Materials and Growth Conditions

The *LIL1* mutant was derived from an M_1_ population of the indica rice cultivar 93-11 after treatment with 0.1% EMS. The leaf lesion phenotype was genetically stable for more than four generations under greenhouse and field conditions. Therefore, the F_1_ hybrid and F_2_ or BC_1_ progenies derived from crossing *LIL1* with TeQing or Nipponbare were used for genetic analysis and molecular mapping. All the plants were grown at the Hunan Agricultural University in Changsha, or at Lingshui, Hainan, China.

To determine the occurrence of the lesion phenotype under aseptic conditions, the seeds of wild-type (WT) 93-11 and *LIL1* were surface sterilized for 30 min in 20% bleach, rinsed three times in sterile water, and germinated in autoclaved MS medium in a 1000 mL glass cylinder at 25°C and with 14 h light/10 h dark.

### Trypan Blue and DAB Staining

Trypan blue staining is a histochemical method to detect cell death or irreversible membrane damage. Leaves of *LIL1* were harvested for trypan blue staining when visible lesions were apparent. Trypan blue staining was performed as previously described (Bowling et al., 1997).

3,3′-diaminobenzidine (DAB) staining was used to detect H_2_O_2_ accumulation in the leaves as previously described (Wang et al., 2007). Leaves of *LIL1* were collected for the DAB staining prior to the appearance of macroscopic lesions.

### Evaluation of Rice Blast Resistance

The *LIL1* mutants were evaluated for blast resistance following manual inoculation in a growth chamber. The seedlings of *LIL1* and 93-11 were grown in plastic trays filled with fertile soil in a greenhouse at 27–30°C. A 5 μL droplet of a spore suspension of *M. grisea* (∼2.0 × 10^5^ conidia/mL) was placed onto the leaf blades of 4 week old seedlings followed by incubation in a growth chamber for 24 h in the dark at 26–27°C. Following inoculation, the leaf blades were misted with water after every 6-7 h for 4–5 days to maintain moisture and facilitate infection. The disease symptoms were observed after 7 days after inoculation.

### RT-qPCR and Semi-quantitative RT-PCR Analysis

Total RNA was extracted with Trizol reagent (Invitrogen Biotechnology Co., Shanghai, China) according to the protocol. After DNaseI treatment, 1 μg of RNA was added to a 20 μL reaction system to synthesize first-strand cDNA using the Reverse Transcription System (Invitrogen Biotechnology Co., Shanghai, China) according to the instructions. Relative quantitative reverse transcription polymerase chain reaction (RT-qPCR) to assess the transcript abundances of defense-related genes were performed in 25 μL reactions using 1.0 μL of 1:10 diluted cDNA as template with 3 technical replicates within each biological replicate. Semi-Quantitative RT-PCR on candidate genes within the *LIL1* mapping interval and on Nipponbare lines over-expressing LOC_Os07g30510 from *LIL1* were performed in 25 μL reactions using 1.0 μL cDNA as template. In each case, the rice actin gene was used as the internal control. All primers for RT-PCR are list in **Supplementary Table S1**.

### Genetic Analysis of *LIL1*

In November 2012, F_1_ hybrids, BC_1_ or F_2_ progenies derived from the reciprocal crosses between 93-11 and *LIL1*, TeQing and *LIL1*, Nipponbare and *LIL1* were planted in Lingshui, Hainan Province. Segregation ratios of the leaf lesions were examined in the F_2_ and BC_1_ progenies at the booting stage.

### DNA Extraction and SSR Analysis

Total rice genomic DNA from both parents and each individual of the F_2_ was extracted from mature leaves as described by McCouch et al. (1988). Microsatellite (simple sequence repeats, SSR) primer sequences were adopted from Temnykh et al. (2000) and McCouch et al. (2002) and synthesized by Invitrogen Biotechnology Co., Shanghai, China. The basic SSR procedure was as follows: 10× reaction buffer 2.0 μL, 2.0 mM of each dNTP, 2.0 U of *Taq* polymerase, 50 ng of template DNA, 30 ng of each primer, and distilled water in a 20 μL reaction that was overlaid with a drop of mineral oil. The amplification reactions were performed as follows: 95°C for 5 min; 30 cycles of 95°C for 30 s, 55°C for 30 s, 72°C for 30 s; and 72°C for 10 min. All PCR reactions were performed in an MJ PTC-100 thermocycler (Waltham, MA, United States). The amplification products were observed in a 8% polyacrylamide gels or 5% agarose gels stained with silver or ethidium bromide, respectively.

### Development of New Markers

Simple sequence repeats sequences in BACs near the target gene were identified using SSRHunter (Li and Wan, 2005). CAPS markers were obtained according to the rice DNA polymorphic database between Nipponbare (*O. sativa* ssp. *japonica*) and 93-11 (*O. sativa* ssp. *indica*) (Shen et al., 2004). Primer Premier 5.0 was used to design all of the primers. Those markers that exhibited polymorphisms between TeQing and *LIL1* were applied in the F_2_ mapping population.

### Data Analysis and Linkage Mapping

The molecular data were analyzed by MAPMAKER/EXP 3.0b (Lander et al., 1987). The band types identical with that of TeQing, *LIL1*, and F_1_ were recorded as 1, 2, and 3, respectively. MAPDRAW2.1 (Liu and Meng, 2003) was used to a construct the linkage map.

### Gene Annotation and Sequence Analysis

The candidate region was annotated by the Rice Genome Annotation Project^1^ in *japonica* rice Nipponbare. The candidate genes were sequenced by the Shanghai Invitrogen Biotechnology Co., China. PCR reactions were performed using KOD-Plus-Neo *Taq* polymerase (TOYOBO life science, Japan) and amplified fragments were separated on a 1.0% agarose gels. ContigExpress was used to assemble sequences and DNAMAN 5.2.2 was used to align the sequences.^2^

### Mutant Validation

To confirm the role of LOC_Os07g30510 in the mutant phenotype of *LIL1*, we cloned the coding sequence of LOC_Os07g30510 from *LIL1* by PCR amplification with gene-specific primers (5′-CGGAATTCATGGCCATTTTGACCGTTCTG-3′ and 5′-CTGTCTAGATTACCTGCCGTTCACAATTGTT-3′). Underlining indicates EcoRI and XbaI sites in the forward and reverse primers, respectively. LOC_Os07g30510 from *LIL1* was cloned into the EcoR1 and Xba1 sites downstream from the rice Actin1 promoter in the binary vector pCAMBIA2300 and transformed into Nipponbare by *Agrobacterium tumefaciens*-mediated transformation. Primers for identifying the transgenic plants were 5′-GAATCCCTCAGCATTGTTC-3′ and 5′-TCACGAGGGTGACGAAGT-3′, with annealing sites on the rice Actin1 promoter and LOC_Os07g30510, respectively.

## Result

### Phenotypes of *LIL1* Mutant

**FIGURE 1:**
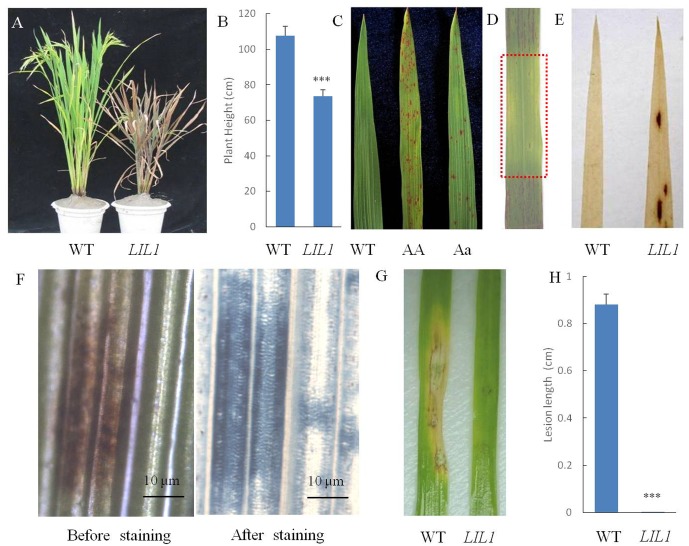
Phenotype characterization of the *LIL1* mutant. **(A)** Photographs of representative plants of the WT (right) and *LIL1* mutant (left) at filling stage; **(B)** Plant height of WT and *LIL1*. Values are means ± standard errors of 10 replications. Significance was determined at ^∗∗∗^*P* < 0.0001 with a Student’s *t*-test; **(C)** *LIL1* is partially dominant: 93-11 (WT), *LIL1* Homozygous (AA), *LIL1* Heterozygous (Aa); **(D)** Determination of whether the *LIL1* phenotype was light dependent. The area covered with aluminum is foil boxed with a red rectangle; **(E)** DAB staining results; **(F)** Trypan blue staining results. **(G)** Disease phenotypes of WT and *LIL1* after inoculation with *M. grisea* isolate ZB25; **(H)** Lesion length of WT and *LIL1* after inoculation with ZB25. Values are means ± standard errors of 10 replications. Significance was determined at ^∗∗∗^*P* < 0.0001 with a student’s *t*-test.

Compared with the WT, the plant height of the *LIL1* mutant was reduced significantly (**Figures 1A,B**). Lesions of the *LIL1* mutant first appeared on leaf tips at approximately the 3-leaf stage and then expanded to the whole leaf surface and eventually the leaf sheath. The lesions were small and rust red in color. The older leaves with many lesions turned yellow and showed early senescence. Ultimately, the whole *LIL1* plants dried up earlier than the WT plants (**Figure 1A**). The lesion formation was controlled by a semi-dominant gene (**Figure 1C**), which decreased remarkably when light was intercepted by covering the leaf with aluminum foil (**Figure 1D**). To determine whether lesion formation in *LIL1* required an exogenous biotic trigger, we grew *LIL1* under sterile conditions. The initiation of lesion in these mutants was the same as plants grown in greenhouse conditions (data not shown). The result suggests that the lesion formation in *LIL1* mutant is not caused by other biotic agents. To determine if H_2_O_2_ accumulation is associated with the onset of lesions, we conducted DAB staining on leaf tips from 2 weeks old plants that did not yet show any visible lesions. Brownish spots appeared on the pre-symptomatic leaves of *LIL1* indicating that there is a burst of active oxygen species that precedes the onset of visible lesions (**Figure 1E**). To determine if the lesions on *LIL1* are associated with plant cell death, we conducted trypan blue staining on lesioned leaves from 3.5 weeks old plants. The appearance of deep blue staining at the lesion sites in *LIL1* mutants indicates the occurrence of extensive cell death (**Figure 1F**).

### Enhanced Resistance of *LIL1* Mutant to Rice Blast Pathogens

Many LMMs exhibit enhanced resistance to pathogens. To determine whether the mutation of *LIL1* led to enhanced resistance to pathogens, we inoculated 4-week-old *LIL1* and 93-11 plants with *M. grisea* isolates RB2, RB17, RB13 and ZB25. While 93-11 and *LIL1* did not show a difference in resistance to *M. grisea* isolates RB2, RB17 or RB13 (data not shown), there was a significant difference in resistance to *M. grisea* isolate ZB25. The rice blast symptoms caused by ZB25 on leaves of *LIL1* were almost invisible in stark contrast to the strong symptoms apparent on 93-11 (**Figures 1G,H**). Thus, *LIL1* plants display significantly increased resistance against *M. grisea*.

**FIGURE 2:**
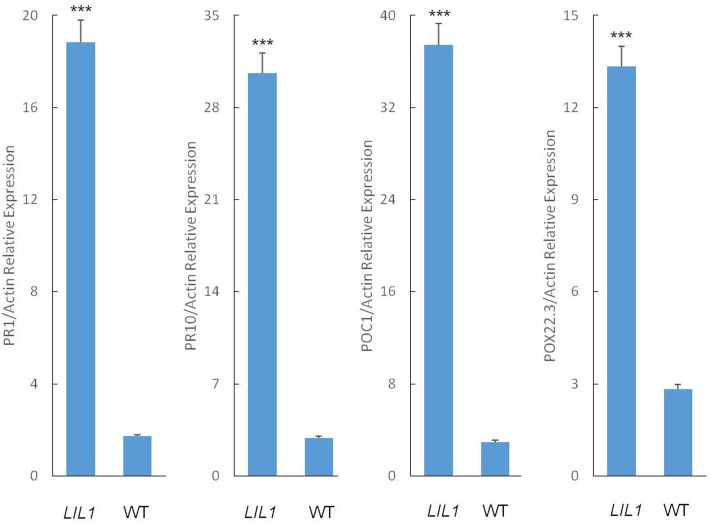
Defense-related gene expression in the *LIL1* mutant. Transcript abundance was determined via RT-qPCR. The error bars represent the standard deviation between three biological replicates. Mean fold-changes in the transcript abundance were calculated using the ΔΔCt method between biological replicates ± standard deviation. Significance was determined at ^∗∗∗^*P* < 0.0001 with a student’s *t*-test.

In plants with enhanced disease resistance, defense genes frequently are expressed constitutively at elevated levels. To determine whether this is the case in *LIL1*, we analyzed the expression of pathogenesis-related (PR) genes and peroxidase genes in the flag leaf of *LIL1* plants at the filling stage using quantitative RT-PCR. Consistent with the enhanced disease resistance of *LIL1*, the transcripts of the PR genes, *PR1* and *PR10*, and the peroxidase genes, *POC-1* and *POX22.3*, were significantly increased in *LIL1* compared to 93-11 (**Figure 2**).

### Genetic Analysis

The *LIL1* mutant was inter-crossed with two *indica* rice cultivars (TeQing, 93-11) and a *japonica* cultivar (Nipponbare). The F_1_ hybrid, BC_1_ and F_2_ populations were obtained from the reciprocal crosses between 93-11 and *LIL1*, TeQing and *LIL1*, Nipponbare and *LIL1*. All of the F_1_ hybrids exhibited lesions on the leaves but, consistent with the semi-dominant nature of the mutation, lesioning in the F_1_ hybrids was significantly less severe than in *LIL1* homozygotes (data not shown). The segregation ratios in the F_2_ and BC_1_ were fitted to 3:1 and 1:1 (**Table 1**). These results indicated that the lesion mimic phenotype was controlled by a semi-dominant gene and not affected by the cytoplasm.

**Table 1:** Segregation of the lesion trait in the progenies of *LIL1.*

Cross	*F*_1_		*F*_2_(3:1)		BC_1_(1:1)			
	Lesion	Normal	Lesion	Normal	Lesion	Normal	χ^2^	*P*
93-11 ×*LIL1*	11	0	147	51			0.05	0.75–0.90
TeQing ×*LIL1*	34	0	1307	451			0.38	0.50–0.75
Nipponbare ×*LIL1*	37	0			82	94	0.82	0.25–0.50

A mapping population consisting of 67 dominant individuals and 45 SSR markers distributed on different chromosomes were used to analysis the linkage relation between markers and the *LIL1* locus. From the TeQing ×*LIL1* cross, the “TeQing” homozygote, heterozygote, and “*LIL1*” homozygote segregation ratios of markers on chromosome 7 (RM1243, RM5481, and OSR22) were 4:37:26, 1:38:28, and 2:36:29, respectively, indicating that the *LIL1* locus is located on the rice chromosome 7.

**FIGURE 3:**
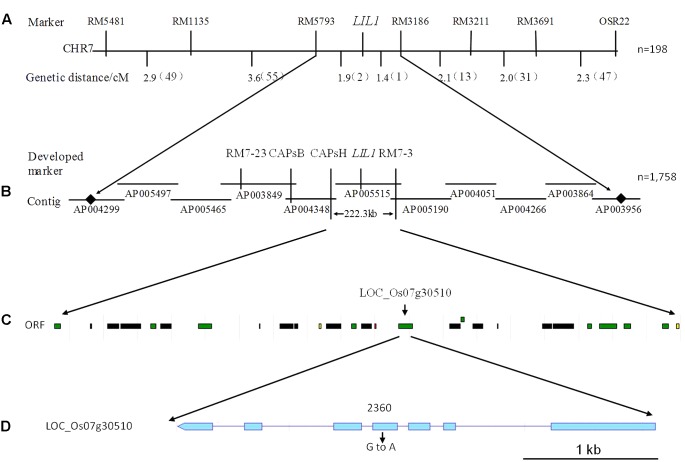
Fine mapping of the *LIL1* gene. **(A)** Primary mapping of the interval between the RM5793 and RM3186 markers on chromosome 7 from *LIL1*; **(B)** Fine mapping of the interval between CAPs H and RM7-3 markers from *LIL1*; **(C)** Putative ORFs predicted in the interval between CAPs H and RM7-3. Green boxes indicate predicted proteins, black boxes indicate transposon or retrotransposon proteins, yellow boxes indicate expressed proteins and red boxes indicate hypothetical proteins; **(D)** Structure of the candidate gene for *LIL1*. Boxes indicate exons and lines indicate introns.

Based on these results, we used additional markers on chromosome 7 to more finely map the *LIL1* locus. First, using 198 individuals from the F_2_ population and seven pairs of polymorphic SSR markers detected from 89 pairs of SSR markers published on a website^3^, the *LIL1* gene was mapped between RM5793 and RM3186 (**Figure 3A**). And then, two SSR markers and two CAPS markers developed by the laboratory (**Supplementary Table S2**) and more 1,758 individuals from the F_2_ population were used for mapping. Finally, the *LIL1* gene was mapped in a 222.3 kb region between CAPS H and RM7-3 on the long arm of chromosome 7 (**Figure 3B**).

### Candidate Gene for *LIL1*

**FIGURE 4:**
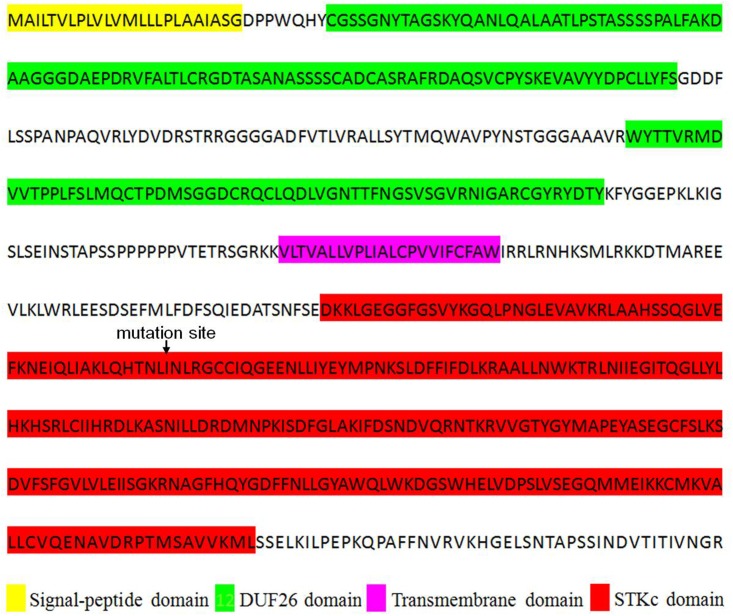
Deduced amino acid sequence of LIL1 protein kinase. Protein domains, indicated by highlighted colors, are defined below the protein sequence.

The Rice Genome Annotation Project^4^ predicts 12 ORFs within the *LIL1* mapping interval (**Table 2** and **Figure 3C**). Sequencing these ORFs revealed that a base G was substituted with A at 2360-nt into the genomic sequence for LOC_Os07g30510, whereas no base changes occurred in the other genes. LOC_Os07g30510 is predicted to encode a 687 amino acid CRK protein consisting of a signal-peptide domain, two domains of unknown function 26 (DUF26), a transmembrane domain, and a STKc domain, the latter of which contains the mutated amino acid (Val 429/Ile) in *LIL1* (**Figure 4**). To test whether the mutation is induced by ethylmethane sulfonate, 10 individuals of *LIL1*, 93-11 and 10 other varieties, including five cultivated rice and five wild rice, were sequenced at this site. All *LIL1* individuals have an A and all others have a G at nt 2630 of LOC_Os07g30510 (**Supplementary Figure S1**). The expression levels of 12 ORFs of *LIL1* and 93-11 were compared and significant differences were not observed for genes other than LOC_Os07g30510 (data not shown). However, the expression level of LOC_Os07g30510 was up-regulated significantly in *LIL1* (**Supplementary Figure S2**). These results focused our attention on LOC_Os07g30510 as the candidate gene for *LIL1*.

**Table 2:** Predicted genes in the mapped region (222.3 kb).

Gene name	Predicted function
LOC_Os07g30330	Cytokinin-*O*-glucosyltransferase 2, putative, expressed
LOC_Os07g30369	Indole-3-acetate beta-glucosyltransferase, putative, expressed
LOC_Os07g30410	DUF26 containing cysteine-rich receptor kinase, putative, expressed
LOC_Os07g30450	Expressed protein
LOC_Os07g30469	Indole-3-acetate beta-glucosyltransferase, putative, expressed
LOC_Os07g30510	DUF26 containing cysteine-rich receptor kinase, putative, expressed
LOC_Os07g30540	Variant latency associated nuclear antigen, putative, expressed
LOC_Os07g30590	Protease inhibitor, putative, expressed
LOC_Os07g30600	Male sterility protein, putative, expressed
LOC_Os07g30610	Cytokinin-*O*-glucosyltransferase 2, putative, expressed
LOC_Os07g30620	Cytokinin-*O*-glucosyltransferase 2, putative, expressed
LOC_Os07g30630	Expressed protein

### Mutant Validation

**FIGURE 5:**
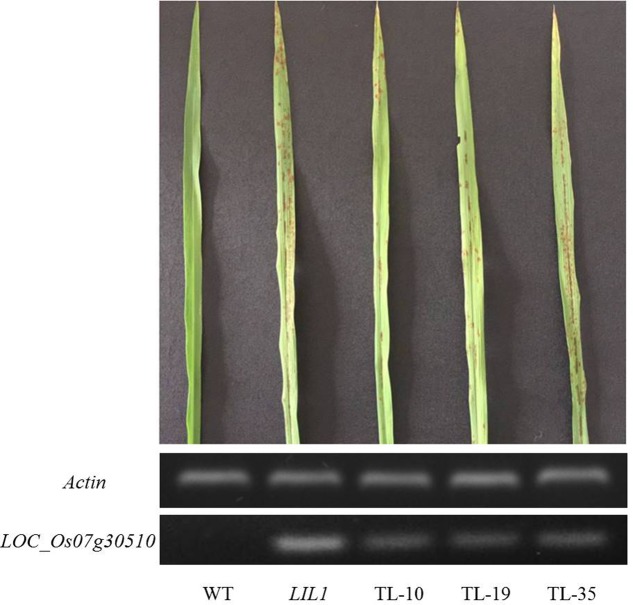
Induction of a lesion mimic phenotype in Nipponbare by over-expression of the *LIL1* mutant-type of LOC_Os07g30510. TL-10, TL-19, and TL-35 are T_0_ plants of three independent transgenic lines in which LOC_Os07g30510 from *LIL1* is expressed under control of the *Actin1* promoter. Phenotypes are representative of an additional 18 lines.

We sought to confirm that LOC_Os07g30510 is the *LIL1* mutant. Given that overexpression of LOC_Os07g30510 in the mutant may account for the phenotype, we surmised that a traditional complementation experiment, in which the WT gene is added to the mutant, would unlikely be informative. Instead, we asked if overexpression of the mutant allele of LOC_Os07g30510 from *LIL1* was sufficient to cause the lesion mimic phenotype in an otherwise WT background. The full-length cDNA sequence of LOC_Os07g30510 from *LIL1* was inserted into vector pCAMBIA2300 under control of the rice Actin1 promoter and was transformed into Nipponbare by Agrobacterium-mediated transformation. Of 32 regenerated T_0_ plants, 21 were positive transformants and each of those 21 exhibited the mutant phenotype (e.g., three transgenic lines shown in **Figure 5**). The transgenic Nipponbare lines exhibited the mutant phenotype later than *LIL1*, with lesions first observed at the booting stage compared to the three-leaf stage in *LIL1*. Semi-quantitative RT-PCR indicated that the tested transgenic lines over-expressed LOC_Os07g30510 relative to Nipponbare, where expression was not detected (**Figure 5**). Expression of LOC_Os07g30510 in the transgenic lines was slightly less than in the *LIL1* mutant, possibly accounting for the delayed onset of lesions in the trangenics. Therefore, we confirmed that over-expression of the mutant form of LOC_Os07g30510 from *LIL1* is sufficient to cause the lesion mimic phenotype of *LIL1*.

## Discussion

Plants have developed complicated signaling pathways and defense mechanisms to protect themselves against invasion of pathogens. The HR is an output of many of these pathways and a component of effective defense against biotrophic and hemi-biotrophic pathogens. However, the molecular mechanisms regulating the HR remain largely unknown (Hunt et al., 1996). LMMs, which include a broad group of phenotypes displaying spontaneous cell death in the absence of biotic challenge, are interesting genetic materials for dissecting the pathways of HR and disease resistance. Lesion appearance in these mutants differs in timing, induction conditions, extent of lesion spreading, color and size (Yin et al., 2000). So far, dozens of genes that control the lesion mimic phenotype have been cloned, and the functions of these genes fall into various groups (Dietrich et al., 1997; Yamanouchi et al., 2002; Balague et al., 2003; Zeng et al., 2004; Qiao et al., 2010; Chen et al., 2012; Fekih et al., 2015; Li et al., 2016; Wang et al., 2017). These results indicate that lesion mimic phenotypes are regulated by different biological processes, thus hinting at the complexity of molecular mechanisms and signaling networks involved in HR and disease resistance. (Lorrain et al., 2003).

Spontaneous lesion formation in the *LIL1* mutant correlates with the expression of disease resistance response genes. The induction of *PR* genes is strongly correlated with the initiation of systemic acquired immunity in both dicot and monocot LMMs (Dietrich et al., 1994; Morris et al., 1998; Takahashi et al., 1999). The *PR1* gene is induced in the rice blast resistance reaction (Schweizer et al., 1998). The *PR10* gene, which encodes an intracellular protein, is induced by the chemical probenazole (3-allyloxy-1,2-benzisothiazole-1,1-dioxide) that also induces host resistance against rice blast (Midoh and Iwata, 1996). Indeed, the *PR1* and *PR10* were highly expressed in the *LIL1* mutant relative to WT plants (**Figure 2**). Consistent with the enhanced expression of *PR1* and *PR10*, the *LIL1* mutant did display enhanced resistance to one of four tested isolates of *M. grisea*. The lack of phenotypic difference between *LIL1* and 93-11 with the other three isolates may result from the strong disease resistance of 93-11 against those isolates. The generation of active oxygen species is an important step during plant-pathogen interactions (Baker and Orlandi, 1995; Low and Merida, 1995). Peroxidases (EC1.11.1.7, H_2_O_2_ oxidoreductase) catalyze the oxidation of various inorganic and organic substrates at the expense of H_2_O_2_, which is both a signal molecule and executor of many plant environmental and developmental responses (Gaspar et al., 1982). Chittoor et al. (1997) reported the induction of rice peroxidases *POX22.3* and *POC1* is correlated with the resistant interactions between rice and the bacterial blight strain *Xanthomonas oryzae* pv. *oryzae*. The *POX 22.3* and *POC1* genes were highly activated in *LIL1* relative to WT plants (**Figure 2**), although the mutant and WT plants did not differ in their resistance to *X. oryzae* in our tests (data not show).

So far, at least 37 of the 49 rice LMMs have been reported to enhance resistance to at least one pathogen (Huang et al., 2011). However, the details of the underlying mechanism of HR-like lesion mimic formation and the resulting specificity of disease resistance remains poorly understood. In the present study, we cloned a rice LMM, *LIL1*, which exhibited spontaneous cell death without pathogen attack. Genetic analysis and molecular mapping indicated that *LIL1* was controlled by a semi-dominant gene located on the long arm of chromosome 7. To our knowledge, this is the first rice LMM semi-dominant gene mapped on chromosome 7. Therefore, the *LIL1* mutant is a novel LMM of rice. Moreover, most of the LMMs are controlled by recessive genes (Yin et al., 2000; Yamanouchi et al., 2002; Qiao et al., 2010; Wang et al., 2015), only a few mutations are regulated by dominant genes (Mori et al., 2007; Wang et al., 2016). In the homozygous *LIL1* mutant, the lesions were much more abundant than those in the heterozygous, indicating that the lesion mimic phenotype of *LIL1* was controlled by a semi-dominant gene. The *LIL1* mutant was mapped to a 222.3 kb region containing 12 putative ORFs on the long arm of chromosome 7. Sequencing these 12 ORFs revealed one base substitution mutation (G/A) (**Figure 3D**) leading to an amino acid change (Val 429/Ile) in the ORF of LOC_Os07g30510. The mutation was observed in 10 more *LIL1* individuals and not observed in 10 individuals of 93-11. Moreover, an additional five cultivated and five wild rice varieties showed the sequence matching to 93-11, indicating that this site is evolutionarily conserved. The expression level of LOC_Os07g30510 was up-regulated significantly in *LIL1*; however, expression of the other 11 ORFs in our mapping interval showed no obvious difference between *LIL1* and 93-11. LOC_Os07g30510 encodes a predicted cysteine-rich repeat kinase (CRK), which is a member of a sub-family characterized by one or more extracellular DUF26 domains containing a C-X8-C-X2-C motif. The DUF26 domain is known as stress-antifungal domain (PF01657) (Sawano et al., 2007). Over-expressing the mutant-type gene of LOC_Os07g30510 is sufficient to cause the lesion mimic phenotype in transgenic lines. Therefore, the up-regulated expression and/or the point mutation in the kinase domain of LOC_Os07g30510 is likely responsible for *LIL1* phenotype. Both overexpression of CRKs and the point mutation in the kinase domain could result in the HR-like cell death and increased plant resistance observed. Overexpression of *AtCRK5, AtCRK13* and *AtCRK20* from Arabidopsis leads to cell death and increased resistance to the bacterial pathogen *Pseudomonas syringae* (Chen et al., 2003, 2004; Acharya et al., 2007; Ederli et al., 2011). Further, a missense mutation on an Arabidopsis RLK gene (*snc4-1D*) at the kinase domain increases its expression and results in enhanced pathogen resistance (Bi et al., 2010). The *snc4-1D* is a gain-of-function semidominant mutant gene, similar to *LIL1.* It is currently unclear whether the phenotype of *LIL1* is due to overexpression of LOC_Os07g30510 or the point mutation in the kinase domain. The point mutation could be the binding region for a microRNA or non-coding RNA, which results in the accumulation of *LIL1* transcript. Based on this hypothesis, the region was used to search the microRNA database “miRbase” and “psTarget” and the non-coding RNA database “NONCODE” and “lncRNAdb”; however, no match was found. In WT plants, microRNA or non-coding RNA may bind and degrade the *LIL1* transcript, this may explain why the *LIL1* transcript could not be detected in WT plants, and potentially why there was no match in the four RNA databases. Further work needs to be performed to confirm this hypothesis. Functions of *LIL1* in HR regulation and disease resistance also need to be clarified in future work.

## Conclusion

Here, we identified and cloned a rice LMM *LIL1* from an ethylmethane sulfonate mutagenized population of *Indica* rice (*Oryza sativa* L. ssp. *Indica*) 93-11. Characterization and molecular mapping revealed that *LIL1* is a novel rice LMM with enhanced resistance to rice blast fungus (*M. grisea*). Genetic analysis illustrated that the mutation was controlled by a semi-dominant gene. Using 1,758 individuals in the F_2_ progenies, the mutant gene was located in a 222.3 kb region on the long arm of chromosome 7. The candidate genes on the interval were sequenced, and it was found that a G for A substitution in the fourth exon of LOC_Os07g30510 led to an amino acid change (Val 429 to Ile) in the serine/threonine kinase catalytic domain of the encoded protein. LOC_Os07g30510 was determined also to be over-expressed in *LIL1* mutant plants. Rice lines transgenically over-expressing the mutant-type gene of LOC_Os07g30510 exhibit a lesion mimic phenotype similar to the *LIL1* plants. Therefore, over-expression of LOC_Os07g30510 and/or the Val429Ile substitution is responsible for the *LIL1* phenotype. Our results provide the basis for additional function analysis of this gene to advance broad understanding of mechanisms underlying HR and disease resistance in rice.

## Author Contributions

QZ and XX conceived and designed the research. QZ collected samples, generated experimental data, performed the entire data analysis, and drafted earlier versions of the manuscript. ZZ and BG partially revised the manuscript. ZZ and TL was involved in the sample collection. All authors read, reviewed, and approved the final manuscript.

## Conflict of Interest Statement

The authors declare that the research was conducted in the absence of any commercial or financial relationships that could be construed as a potential conflict of interest.

## Footnotes

1 http://rice.plantbiology.msu.edu/

2 http://www.lynnon.com

3 http://www.gramene.org

4 http://rice.plantbiology.msu.edu

## Supplementary Material

FIGURE S1 Comparison of the mutation sites at 10 additional WT and *LIL1* individuals as well as 10 other rice varieties. 93-11 is WT, *LIL1* is mutant, Guichao 2, Minghui 63, Weiyou 402, Shanyou 46, Zhonghua 11 are cultivated rice and Ledong, Gaozhou, Dongxiang, Chaling, Yuanjiang are wild varieties of rice.

FIGURE S2 LOC_Os07g30510 expression comparison in WT and *LIL1.* WT is 93-11, AA is *LIL1* Homozygous, Aa is *LIL1* Heterozygous.

TABLE 1 Details of the genes and primer sequences used.

TABLE 2 Polymorphic markers developed on chromosome 7 of rice.
