# Supplementary figures and images for: Identification and Map-Based Cloning of the Light-Induced Lesion Mimic Mutant 1 (LIL1) Gene in Rice
Source: Front Plant Sci. 2017 Dec 19;8:2122. doi: 10.3389/fpls.2017.02122 (PMC5742160; doi:10.3389/fpls.2017.02122)

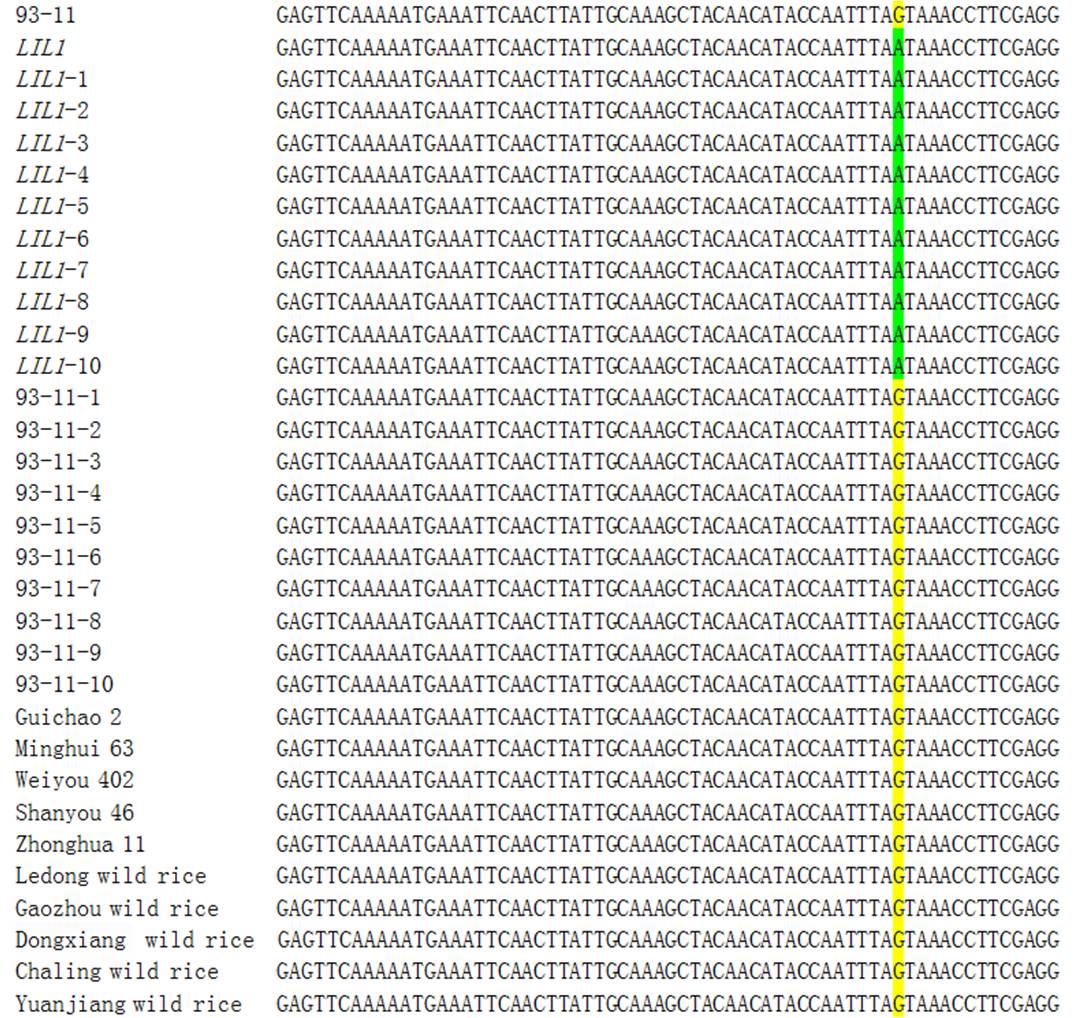

Supplement: FIGURE S1 — Comparison of the mutation sites at 10 additional WT and LIL1 individuals as well as 10 other rice varieties. 93-11 is WT, LIL1 is mutant, Guichao 2, Minghui 63, Weiyou 402, Shanyou 46, Zhonghua 11 are cultivated rice and Ledong, Gaozhou, Dongxiang, Chaling, Yuanjiang are wild varieties of rice. [file Image_1.JPEG]

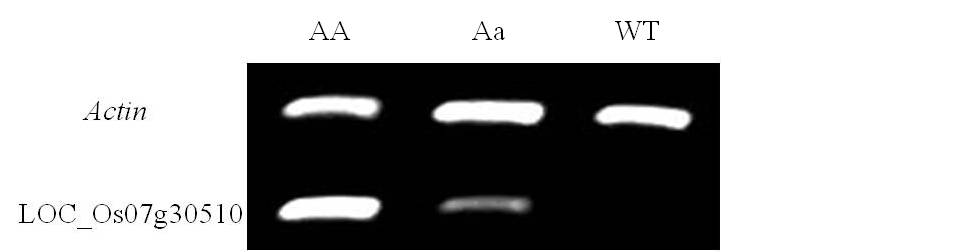

Supplement: FIGURE S2 — LOC_Os07g30510 expression comparison in WT and LIL1. WT is 93-11, AA is LIL1 Homozygous, Aa is LIL1 Heterozygous. [file Image_2.JPEG]
