# Supplementary material for: Identification and Map-Based Cloning of the Light-Induced Lesion Mimic Mutant 1 (LIL1) Gene in Rice
Source: Front Plant Sci. 2017 Dec 19;8:2122. doi: 10.3389/fpls.2017.02122 (PMC5742160; doi:10.3389/fpls.2017.02122)
Supplement: TABLE 1 — Details of the genes and primer sequences used. [file Table_1.DOCX]

Supplementary TABLE 1 | Details of the genes and primer sequences used.

| Gene name | Forward primer (5'-3') | Reverse primer (5'-3') |
| --- | --- | --- |
| *Actin* | TGTAAGCAACTGGGATGA | CCTTCGTAGATTGGGACT |
| *PR1* | CAGGACTACGTGAGGCTCCA | CCTCTGCCGACGAAGTTGC |
| *PR10* | ACCATCTACACCATGAAGCTTAAC | GTATTCCTCTTCATCTTAGGCGTA |
| *POC1* | CATTGCTAGGCAGAGATAAACG | ATGAGACGGTCTGATTGCACAC |
| *POX22.3* | TCGGCGACGTTCTATGACAC | CGYYGTCGATGACGCTGAAT |
| *LOC_Os07g30330* | TCACCGACCAGCAGATCAAC | TCCATCGCCTCCCTTACCAT |
| *LOC_Os07g30369* | ACCGAGGACCAGAACGACC | CGGAGCACCCAGAGGAAT |
| *LOC_Os07g30410* | CAGCGGCAACTACACGG | AGAGGGTCAGGGCGAAC |
| *LOC_Os07g30450* | AAGCGACGGGCAAGAC | AACGCCTCCAAGGAGATAA |
| *LOC_Os07g30469* | ACCGAGGACCAGAACGACC | CGGAGCACCCAGAGGAAT |
| *LOC_Os07g30510* | AAGCAGCGGCAACTACACG | AGAGGGTCAGGGCGAACAC |
| *LOC_Os07g30540* | GGGTGGCGGCATACTTG | CCGTCCGTCCATCGACTT |
| *LOC_Os07g30590* | TGCCCACGCTCTTGATG | TGGTGAAGGCACATTGCA |
| *LOC_Os07g30600* | AAGGCAATGGGTGAGAT | CTTGGCGTAAGCAGAAA |
| *LOC_Os07g30610* | GGGAGCCTCACCGTCATCT | TGCTGCGACAGAGCTTGTG |
| *LOC_Os07g30620* | TTGCTCGCCTCGCTTGT | GACCTCGTTCACCGTTTGC |
| *LOC_Os07g30630* | ACGCCAACAAGAAGATG | GCCAGTGTAAACCGAAA |
