# Supplementary material for: Identification and Map-Based Cloning of the Light-Induced Lesion Mimic Mutant 1 (LIL1) Gene in Rice
Source: Front Plant Sci. 2017 Dec 19;8:2122. doi: 10.3389/fpls.2017.02122 (PMC5742160; doi:10.3389/fpls.2017.02122)
Supplement: TABLE 2 — Polymorphic markers developed on chromosome 7 of rice. [file Table_2.DOCX]

**Supplementary TABLE 2 | Polymorphic markers developed on chromosome 7 of rice**

| **Molecular marker** | | **Primer sequence** | **GenBank No.** | **Restriction enzyme** |
| --- | --- | --- | --- | --- |
| RM7-23 | 5’-GCAAGGGAAGATGAGATGTG-3’  5’-CGTGATGATGAAGGTGGTGT-3’ | | AP003849 |  |
| CAPs B | 5’-CGAGGCAACAATAGGATACCAC-3’ 5’-GGAGATAAGGTTTCTGCGGTCA-3’ | | AP003849 | BstNI |
| CAPs H | 5’-ATAGGACGCTAGGACTAACTT-3’  5’-GGATATCTGTTACACATGAGC-3’ | | AP004348 | HpyCH4III |
| RM7-3 | 5’-TATGCCACGTACGGCCGATC-3’ 5’-GCCCCTGCTGAATGCTGAAC-3’ | | AP005515 |  |
